# Disability shouldn’t limit accessibility in science

**DOI:** 10.1038/s42003-021-02411-8

**Published:** 2021-07-16

## Abstract

July is Disability Pride Month here in New York, where part of the Communications Biology team is based. To mark this occasion, we are featuring a series of scientist interviews on the Nature Portfolio Ecology & Evolution Community site and wanted to elaborate on our motivations behind this post and our hopes for the future concerning the lived experience of disability in science.

Despite disability becoming an increasingly important part of the conversation in science, oftentimes the physical layout of a lab (or field site) and the design of its instruments can render science literally inaccessible to some. And while the representation of disabled scientists has increased from 2008 to 2018^1^, many scientists still choose not to disclose a disability due to exclusionary practices and entrenched ableism at institutions^2^.

Making space for and improving the experience for people with disabilities […] requires the active investment of all facets of science: labs, institutions, and funding bodies; this includes our own practices as journals and publishers.

Making space for and improving the experience for people with disabilities, neurodiversity, or a mental health condition requires the active investment of all facets of science: labs, institutions, and funding bodies; this includes our own practices as journals and publishers. This practice can take many forms, including an increase in quantitative and qualitative research on the experiences of disabled scientists, increased accessibility of websites, accessibility guidelines at conferences, the adoption of Universal Design in labs^3^, and the continuation of remote working practices initiated at the onset of the COVID-19 pandemic^4^. In line with the rallying mantra “nothing about us without us,” the inclusion of disabled scientists themselves at the head of these conversations is crucial. And while this inclusion and accessibility is important regardless, taking accessibility measures in labs, at conferences, and in the field, benefits science and scientists, regardless of their ability^5^.

These practices are necessary for the ongoing inclusion, engagement, and leadership of disabled people in science, but a first step is redefining public perception of what it means to be a scientist with a disability. It is important to consider that every person’s experience is unique, and the identification as “disabled” intersects with other identities. We hope that, by featuring a few of these researchers and their stories in the accompanying blog post, it can lead to a greater understanding of their experience with a disability in research, and highlight what action needs to be taken in order to better support them and improve their experience in science.
